# Reference genes for gene expression studies in wheat flag leaves grown under different farming conditions

**DOI:** 10.1186/1756-0500-4-373

**Published:** 2011-09-27

**Authors:** Gabriela N Tenea, Adrian Peres Bota, Fernando Cordeiro Raposo, Alain Maquet

**Affiliations:** 1 European Commission, Joint Research Centre (JRC), Institute for Reference Materials and Measurements (IRMM), Retieseweg 111, 2440 Geel, Belgium

## Abstract

**Background:**

Internal control genes with highly uniform expression throughout the experimental conditions are required for accurate gene expression analysis as no universal reference genes exists. In this study, the expression stability of 24 candidate genes from *Triticum aestivum *cv. Cubus flag leaves grown under organic and conventional farming systems was evaluated in two locations in order to select suitable genes that can be used for normalization of real-time quantitative reverse-transcription PCR (RT-qPCR) reactions. The genes were selected among the most common used reference genes as well as genes encoding proteins involved in several metabolic pathways.

**Findings:**

Individual genes displayed different expression rates across all samples assayed. Applying geNorm, a set of three potential reference genes were suitable for normalization of RT-qPCR reactions in winter wheat flag leaves cv. Cubus: *TaFNRII *(ferredoxin-NADP(H) oxidoreductase; AJ457980.1), *ACT2 *(actin 2; TC234027), and *rrn26 *(a putative homologue to RNA 26S gene; AL827977.1). In addition of these three genes that were also top-ranked by NormFinder, two extra genes: *CYP18-2 *(Cyclophilin A, AY456122.1) and *TaWIN1 *(14-3-3 like protein, AB042193) were most consistently stably expressed.

Furthermore, we showed that *TaFNRII, ACT2*, and *CYP18-2 *are suitable for gene expression normalization in other two winter wheat varieties (Tommi and Centenaire) grown under three treatments (organic, conventional and no nitrogen) and a different environment than the one tested with cv. Cubus.

**Conclusions:**

This study provides a new set of reference genes which should improve the accuracy of gene expression analyses when using wheat flag leaves as those related to the improvement of nitrogen use efficiency for cereal production.

## Findings

In the post-genomic era, the development of transcriptomic, proteomic and metabolomic approaches has been proposed to investigate the regulation of plant growth, development and physiology of plant cells and, more recently, as a new tool for food authentication [1,2]. Real-time quantitative reverse-transcription PCR (RT-qPCR) represents one of the most widely used technologies for quantification of mRNA transcription levels, due to its sensitivity, specificity, dynamic range and high throughput capacity [3,4]. RT-qPCR requires normalization of variability using one or several reference gene(s) (known as housekeeping gene), which is one of the most important steps for the correct evaluation of gene expression [5,6].

Usually, the reference genes used in gene expression analysis are chosen for their known or suspected housekeeping roles, but numerous studies revealed that these genes (actins, beta-globulin, 18S rRNA) as such are insufficiently stable during the treatments [5,7,8]. Until now, no gene can be considered as a *universal, reliable reference gene *which is expressed at the same level in all types of plant tissue assayed and is not treatment dependent. For instance, the 18S rRNA gene, one of the most widely used housekeeping genes for normalization, is far from being ideal [9]. It requires the use of total RNA and random primers for the RT-qPCR reaction and is expressed at very high levels. In addition, there can be imbalances in rRNA and mRNA fractions between different samples and 18S is not always constantly expressed in all conditions. Also, the *18S *gene expression levels appear to be affected to a lesser extent by partial RNA degradation than are mRNA expression levels [5,9]. The evidence shows that the transcription level of these genes can vary considerably in response to changes in experimental conditions, and/or tissue type, variety used, etc so none of them can be considered as a universal reference. However, every experimental design, which uses gene expression analysis, should first search for a stable expressed reference genes, which are appropriate for the specific set of experimental conditions and types of tissue used [10].

Recently, a number of papers were reported with regard to the selection of a suitable gene for PCR normalization in yeasts, animals, human and plant systems [7,11-14].

Wheat (*Triticum aestivum *L.) is one of the three most important cereal crops worldwide. There is little doubt that wheat will retain its dominant position in European agriculture due to its adaptability and consumer acceptance. However, it may also need to adapt to face changing requirements notably reducing inputs [15]. A number of projects worldwide are therefore focusing on understanding the processes that determine the efficiency of nitrogen uptake, assimilation, and utilization of nitrogen in order to improve the efficiency of nitrogen recovery in the grain [15,16]. Whilst the physical processes of nitrogen and sulphur remobilization have been studied in detail, the genetic control of these processes and their contribution to agronomic productivity are less well understood [17]. The particular complex structure of the flag leaf allows for an efficient translocation of assimilates until the very late stages of leaf senescence [18], and the relative contribution of the flag leaves to the final grain nitrogen level is essential [19]. In this context, organic farming relies on a number of objectives and principles, as well as common practices designed to minimise the human impact on the environment, while ensuring that the agricultural system operates as naturally as possible. Typical organic farming practices prescribe strict limits on chemical/synthetic pesticide and synthetic fertiliser use [20,21]. In addition, winter wheat is the most demanding cereal in organic food market for bread consumption [22]. On the other hand in conventional agricultural practices there is an increasing interest in fertilisers using nitrogen in solution. Such fertilisers increase the distribution efficiency of nitrogen and permit marginal direct leaf uptake of the dissolved forms of nitrogen.

To our knowledge, transcriptional profiling methods, such as RT-qPCR, in selection and identification of potential discriminative genes in wheat cultivated under organic and conventional environmental conditions have not yet been fully developed. A previous study was carried out regarding the identification of gene markers in wheat grown with organic and inorganic fertilisers [23]. It has been suggested that several genes are differentially expressed in grain endosperm when nitrogen is supplied in both organic and inorganic conditions and these genes are related to nitrogen metabolism and storage protein synthesis.

With regard to the selection of a potential reference gene in wheat, a new *in silico *method based on data publicly available in databases such as UniGene http://www.ncbi.nlm.nih.gov/UniGene and TIGR Gene Indice http://compbio.dfci.harvard.edu/tgi/plant.html has been recently proposed [7]. Expression stability was tested among 32 genes including housekeeping genes and new candidates genes in one wheat variety, and three new reference genes, Ta.54227 (AAA-superfamily of ATP-ases), Ta.2291 (ADP-ribosylation factor), and Ta.2776 (RNase L inhibitor-like protein) were selected as stable reference genes for normalization of gene expression in different tissues and developmental stages of wheat. Even more recently, using genome-wide identification of novel reference genes in the wheat Chinese Spring variety under controlled environmental conditions, Long et al. showed that 15 new candidate genes are better internal controls than traditional reference genes [24]. Nonetheless, none of them correspond to the three reference genes selected by Paolacci et al [7]. These results emphasise the importance to select appropriate reference genes for specific experimental design.

Contrary to the previous studies, we used RT-qPCR gene expression analysis to search for genes which are uniformly expressed in more than one winter wheat variety grown in open field conditions under conventional *vs*. organic farming systems. The expression stability of 24 candidate genes was evaluated by applying two of the available software tools (i.e. geNorm and NormFinder), which have been developed to select the most stably expressed genes to use under given experimental conditions [25,26]. We have selected a set of potential reference genes for normalization of gene expression in winter wheat flag leaves. Further, the expression stability of the selected reference genes in cv. Cubus was evaluated in two additional winter wheat varieties, Tommi and Centenaire, grown under three field conditions: no nitrogen, organic and conventional farming systems.

## Methods

### Plant material and environmental design

Flag leaves (the last leaf before the ear) of winter wheat, *Triticum aestivum*, cv. Cubus were collected in 2006 at the milk stage of ear development in two environments located in the Walloon region of Belgium (i.e. Marloie and Barvaux-en-Condroz). In each environment, certified organic and conventional fields were separated by short distances (i.e. < 5 km) though otherwise located in similar microclimatic conditions with comparable soil properties [27]. A set of 15 flag leaves were collected from each field representing a total of 60 samples (i.e. 2 environments × 2 fields/environment × 15 samples/field). Leaves were sliced longitudinally, submersed in RNA*later*™ buffer (Applied Biosystems/Ambion, Belgium) and stored on ice upon arrival in the lab. The tissue was grinded to powder in liquid nitrogen with a mixer mill MM301 (Retsch, Germany) and the powder stored at -80°C until processing.

Two other varieties, Tommi and Centenaire, grown under three treatments (i.e. no nitrogen fertilisation, organic and conventional agricultural systems) were collected in 2008 at the same developmental stage in one additional environment (i.e. Ciney) of the Walloon Region (Belgium). A similar experimental design was applied except for the number of samples per field of which 30 samples were used (i.e. 5 flag leaves/field × 3 fields/variety × 2 varieties).

All data related to the crop husbandry applied by the farmers during the full study were recorded and available on request.

### Total RNA isolation and cDNA synthesis

Total RNA was isolated from wheat flag leaves (100 mg powder) using a Qiagen RNA isolation kit (Qiagen, Carlsberg, CA, USA) following the manufacturer's instructions. Quality and integrity of RNA was assayed using an Agilent RNA 6000 Nano kit following the manufacturer's protocol (Agilent 2100 Bioanalyzer, cat # 5067-1511). The RNA quality varied between samples and occasionally partial degradation of RNA was observed. For further analysis the RNA integrity number (RIN) was determined using Agilent RNA 6000 Nano kit and the samples with a RIN value higher than 5.5 were used. The RNA concentration was determined using the RediPlate 96 RiboGreen RNA quantification kit (Quant-IT RiboGreen RNA reagent and kit; Invitrogen; cat # R11419). The purity of RNA was estimated not only from the ratio between absorbance readings at 260 nm and 280 nm (suitable range 1.8-2.1) but also from the ratio A_260/_A_230_, which values where higher than 2 indicating neither protein contamination nor other contaminations with reagents used in the extraction. Further, the contaminating genomic DNA was removed by incubation of total RNA (2 μg) with 2 U/μL Turbo DNAseI enzyme (Applied Biosystems/Ambion kit, cat # AM1907) for 30 min at 37°C and the reaction was stopped using a DNAseI inhibitor reagent.

First strand complementary DNA was synthesized using the High-capacity cDNA reverse transcription kit (M-MLV: Moloney Murine Leukemia Virus Reverse Transcriptase, cat # 4374966) according to the manufacture instruction's (Applied Biosystems). Total RNA (2.5 μg) was reverse-transcribed in cDNA using with the same amount of RNA in all samples. The method is based on using the random primers for initiating the cDNA synthesis. The reaction was carried out in a total volume of 20 μL. The advantage of using M-MLV for reverse-transcription is due to the fact that the random hexamers will introduce the least bias in the resulting cDNA. All cDNA samples were diluted 1:5 before used in RT-qPCR analysis.

### Selection of gene sequences: primers and probe design

The wheat reference candidate genes (comprising both known housekeeping and genes from other metabolic pathways) were chosen based on previous gene expression studies and/or by database mining (NCBI; blastx) with heterologous plant (*Arabidopsis*, rice) genes annotated in the NCBI http://www.ncbi.nlm.nih.gov/ or TIGR wheat gene index http://compbio.dfci.harvard.edu/cgi-bin/tgi/gimain.pl?gudb=wheat public databases. Candidate reference genes tested are listed in Tables 1 and 2 with their respective reference(s) where they were described first. The *actin *genes are reported with their unique sequences number (TC, Tentative Consensus) according with TIGR Gene Index. These were chosen based on their homology with other *actin *genes tested for their reference gene performance in other plant systems (e.g. rice, *Arabidopsis*). To identify wheat homologues a "tblastx" (NCBI) was run with the candidate gene sequences on NCBI database using the default settings of the on line program and the full length sequence selected. Using Primer Express (version 3, Applied Biosystems), primers, TaqMan and MGB probes were designed for 24 potential candidate genes (a list of primers and probes are presented in Additional files 1 and 2). Wherever possible, probes were designed to span exon/exon boundaries. The probe sequence was blasted (NCBI; blastn/viridiplantae) to check the homology with expressed sequenced tags (ESTs) or high-throughput genomic sequences (htgs) to avoid any cross-amplification. For some genes where we did not find a homologous gene in *Arabidopsis*, the probe was designed on the 3' UTR of the gene.

**Table 1:** Description of the traditional reference candidate genes tested.

Gene identification^1^	Gene name	tblastx results			Putative function/reference
			
		Best hit (characterised)			
			
		Locus	Description	Score	
AF021243.1	*eIF4B*	NM_101172.2	*Arabidopsis thaliana *translation initiation factor	85.8	Eukaryotic translation initiation factor 4B [64]

AF475127.1	*18S *subunit ribosomal protein	EU972520	*Zea mays *18S ribosomal RNA	94.5	18S subunit ribosomal protein

AY049041.1	*Ta28S*	AF479118.1	*Populus tremuloides *26S ribosomal RNA	614	Ribosomal RNA [65]

AY456122.1	*CYP18-2*	RICCYP2G	*Oryza sativa *(*CYP2*)	334	Cyclophilin A [66]

D13147.1	Elongation factor 1 beta	RICEF1B	*O. sativa *(japonica cultivar-group) mRNA for elongation factor 1 beta'	149	Elongation factor 1 beta [67]

TC234027	Actin II (*ACT2*)	AY305724	*Gossypium hirsutum *actin (*ACT2*)	891	[68]

TC234060	Actin 11 (*ACT11*)	AY305724	*Ricinus communis *actin (*ACT*)	853	[68]

TC247734	Actin 11	AY742219	*Saccharum officinarum *actin	888	[68]

TC248038	Actin I	NM_121018	*Arabidopsis *(*ACT7*)	903	[68]

TC248640	Actin 7	AY305724	*G. hirsutum *actin (*ACT2*)	887	[68]

U76558.1	Alpha-tubulin	Y08490	*Hordeum.vulgare *mRNA for alpha-tubulin 2 (*TUB2*)	714	Alpha-tubulin [69]

U76745.1	Beta-tubulin 2 (*Tubb2*)	AF059288	*Eleusine indica *beta-tubulin 2 (*TUB2*)	1016	Beta-tubulin 2 [70]

U76895.1	Beta-tubulin 4 (*Tubb4*)	NM_001112218	*Z. mays *beta-7 tubulin (*TUBB7*)	977	Beta-tubulin 4 [70]

^1^http://www.ncbi.nlm.nih.gov/UniGene or TIGR Gene Indices http://compbio.dfci.harvard.edu/tgi/plant.html

**Table 2:** Description of a new set of candidate genes tested.

Gene Identification^1^	Gene name	tblastx results			Putative function/reference
			
		Best hit (characterised)			
			
		Locus	Description	Score	
AB042193.1	*TaWIN1*	OSU65958	*O. sativa *14-3-3 protein homolog	571	14-3-3 like protein family [62]

AF244997.1	ADP glucose pyrophosphorylase small subunit	Z48562	*H. vulgare *mRNA for ADP-glucose pyrophosphorylase small subunit	1138	ADP-glucose pyrophosphorylase small subunit [71]

AF251264.1	*Triticum aestivum *ribulose-1,5-bisphosphate carboxylase activase B (*RcaB*)	BLYRCAB	*H. vulgare *rubisco activase (*Rca*B)	576	Rubisco activase [72]

AJ457980.1	*TaFNR1I*	AB035645	*Z. mays *L-FNRII mRNA for ferredoxin	687	Ferredoxin-NADP(H) oxidoreductase [73]

AJ635206.1	Rubisco small subunit (*rbcS*)	HVU43493	*H. vulgare *ribulose-1,5-bisphosphate carboxylase small subunit mRNA	328	Photosynthesis related genes [23]

AL827977.1	*T. aestivum *mitochondrion *rrn26 *gene for rRNA large subunit 26S (Z11889.1)	Z11889	*T. aestivum *mitochondrion rrn26 gene for rRNA large subunit (26S)	150	Similar to mitochondrion rRNA 26S [23]

AY587265.1	*NRT1.1*	AF140606	*O. sativa *nitrate transporter (NRT1)	889	Low affinity nitrate transporter NTR 1.1 [74]

AY727927.1	ADP-glucose pyrophosphorylase small subunit	AF537363	*H. vulgare *subsp. vulgare ADP-glucose pyrophosphorylase small subunit	1054	ADP-glucose pyrophosphorylase small subunit [75]

NP_114282	*CLP *gene for proteolytic subunit of ATP-dependent protease	X54484	Wheat chloroplast *clpP *gene for proteolytic subunit of ATP-dependent protease	1136	[23]

NP_114267.1	*RuBisCO *large subunit	EU492898	*T. aestivum *ribulose-1,5-bisphosphate carboxylase/oxygenase large subunit	759	ribulose-1,5-bisphosphate carboxylase/oxygenase large subunit [76]

X14348.1	ADP-glucose pyrophosophorylase (*WL:AGA.1*)	U66876	*H. vulgare *ADP-glucose pyrophosphorylase large subunit (*blpl*)	541	ADP-glucose pyrophosophorylase [77]

^1^http://www.ncbi.nlm.nih.gov/UniGene

### Quantitative real-time PCR

The total PCR reaction volume was 25 μL comprising: TaqMan^® ^Universal PCR Master Mix (2 ×) 12.5 μL; primers/probe premix (25 ×) 1 μL; diluted cDNA 5 μL (10 ×; approximately 100 ng) and milliQ water. The primers and probes were premixed (100 μM and 20 μM, respectively) to obtain a 25 × stock with final concentrations of 900 nM and 200 nM, respectively. PCR amplification and analysis were achieved using an ABI Prism cycler 7900 (Applied Biosystems). A three steps PCR procedure was used: 50°C for 2 minutes; denaturation by a pre-incubation for 10 minutes at 95°C and amplification of template 60 s at 60°C and 15 s at 95°C for 40 cycles. The reactions were carried out in duplicates in a 384 well plate (pipetting was performed with an automated liquid handling system, Freedom EVOware, Tecan, Mechelen, Belgium). The maximum difference accepted between Cq values of technical replicates was 0.5. All reaction components without template were used as a negative control (NTC). The C_q _value (raw quantification cycle, also known as C_t_, threshold cycle) according to RDML guideline (Real-time PCR Data Markup Language) [10] was determined automatically using the SDS 2.2.2 software (Applied Biosystems, Halle, Belgium).

### Determination of PCR efficiency

The PCR amplification efficiency was determined for each primer combination by the slope of the standard curve obtained by plotting the fluorescence versus given concentration of a mixture of all sample cDNAs (ranging from 1:1 to 1:5000 dilution of the cDNA mixture sample) using the equation: E = 10^(-1/slope) ^-1 [10]. The PCR efficiencies ranged from 1.83 to 2.00. The R^2 ^values are included in Additional files 1 and 2. No signals were detected in no-template and no-RT controls (no reverse-transcription).

### Assessment of reference genes stability

For analysis of gene expression stability we used two widely known Microsoft Excel applets, geNorm [28] and NormFinder [25]. Before running geNorm, relative quantities of all tested candidate reference genes were determined by the number of amplification cycles needed to reach a specific threshold level of detection. C_q _values were converted into relative quantities via the modified delta-delta C_q _method implemented in qbase^PLUS ^[29]. The relative quantities were imported in geNorm software (version 3.5) to assess gene stability [28]. The program geNorm computes the expression stability M of a candidate gene assayed on the average pair-wise variation between all genes included in the experiment. The M-value measure relies on the principle that the expression ratio of two hypothetical ideal control genes is identical in all samples, regardless of the experimental conditions or treatments. The lowest M values are produced by genes with the most stable expression. By stepwise exclusion of the least stable gene and recalculation of the M values, the most stable genes are identified. Finally, a normalization factor (NF) is calculated automatically based on the geometric mean of the expression levels of the best performing reference genes. NF varies between 0.31 and 3.24. The software determines also the optimal number of reference genes necessary for gene expression analysis based on pair-wise variation Vn/Vn+1 between sequential normalization factors containing the reference candidate genes. Based on a cut-off value of Vn/Vn+1 < 0.15, the recommended number of references genes is given by n.

NormFinder algorithm use the ANOVA-based model to estimate inter- and intra-group variation and combines these estimations to provide a direct measure of the variation of expression for each gene [25]. In current study, two groups corresponding to the agricultural production systems (organic vs. conventional) were defined. Genes with the least inter-group variation combined to a low intra-group variations were considered to be stable.

Systat 13 (Erkrath, Germany) was used in order to perform descriptive statistics.

## Results

### Selection of a reference gene for gene expression analysis in winter wheat flag leaves

In this study, the stability of gene expression of several candidate reference genes was performed in order to select the most stable expressed gene which can be further used for normalization of PCR reaction for future gene expression studies in winter wheat flag leaves.

**Figure 1:**
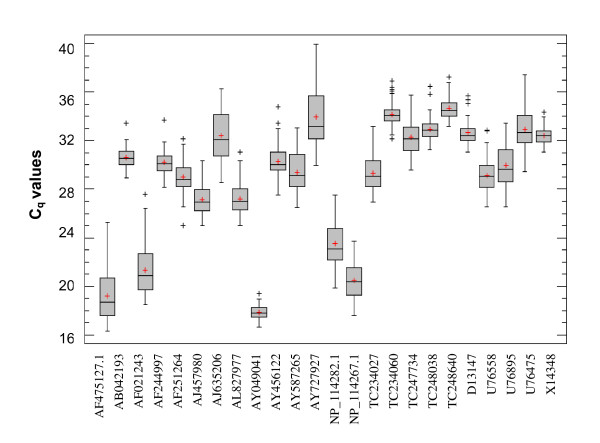
**Gene expression levels (real-time PCR quantification cycle values) in organically and conventionally grown winter wheat cv**. **Cubus flag leaves.** Boxplot represents the median (line), the mean (red cross), 25^th ^to 75^th ^percentile (box), range (whiskers) and outside (black cross) C_q _values.

Analysis of the transcript abundance revealed that the individual candidate reference genes displayed different expression ranges across the full set of wheat samples assayed (Figure 1). While median C_q _values varied from 17.8 with a coefficient of variation (CV) of 3.1% for AY049041 gene to 34.5 with CV of 2.6% for TC248640, most of the genes analysed have shown an expression rate between 26.9 and 30.1. Several genes displayed high expression rates with median C_q _values of 18.7 and CV of 11.1% and 20.4 with a CV of 7.9% for AF475127.1 and NP_114267.1, respectively. Taken together, all the genes assayed displayed a relatively wide range of expression rates in winter flag leaves.

**Figure 2:**
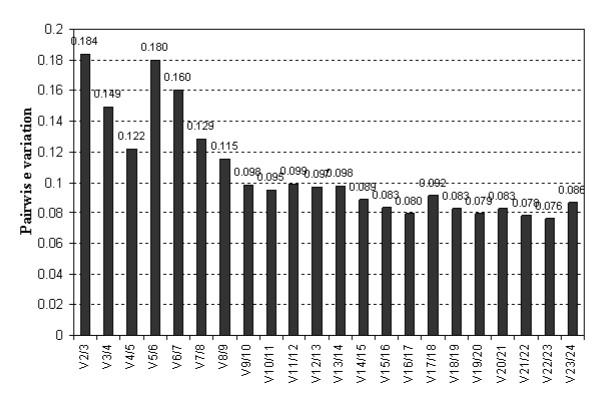
**Determination of optimal number of reference genes based on pair-wise variation (V) analysis of normalisation factors of the candidate reference genes**. A V_n/n+1 _value is shown for every comparison between two consecutive candidate reference genes. As a general guideline it is stated that the benefit of using an extra (n+1)^th ^reference gene is limited as soon as the V_n/n+1 _value drops below the 0.15 threshold.

According to geNorm algorithm, the expression stability (M value) of 24 genes was ranked as presented in Table 3. To evaluate the robustness of ranking genes using geNorm, we performed a bootstrap step as indicated by Gabrielsson et al. (2005) [30] by resampling the genes after exclusion of one of the most stable selected genes and we observed that the remaining gene is in agreement with the initial geNorm ranking (data not shown). Across all the samples of cv. Cubus (60 samples), the lowest M values were obtained for two genes: *TaFNRII *(ferredoxin-NADP(H) oxidoreductase; AJ457980.1), and *ACT2 *(actin 2; TC234027) with M values of 0.5. Other two genes, with accession number AL827977.1 (*rrn26*), with unknown function in wheat but similar with the mitochondrial rRNA 26S unit and AY456122.1, a cyclophilin A, displayed also a M value of 0.57 and 0.62, respectively across the samples tested for the same variety. Pair-wise variation (V = Vn/Vn+1) between consequently ranked normalization factors was calculated by the geNorm program to evaluate the optimal number of genes required for accurate normalization. When all genes were analysed, the pair-wise value for two genes V_2/3 _was 0.184, while the pair-wise value for three genes was 0.149 (Figure 2). Therefore three genes could be included for normalization and be ideal for further gene expression evaluations in organic and conventional winter wheat flag leaves cv. Cubus.

**Table 3:** Candidate genes ranked according to their expression stability as determined by geNorm and NormFinder

geNorm		NormFinder	
**Gene Identification**	**Stability value (M)**	**Gene Identification**	**Stability value**

AJ457980/TC234027	0.50	AB042193	0.155

		AF244997	0.195

AL827977	0.57	TC248640	0.203

AY456122	0.62	AY456122	0.210

AJ635206	0.66	AY049041	0.220

X14348	0.82	AL827977	0.225

AB042193	0.92	AJ457980	0.230

TC248038	0.99	TC234027	0.246

AY049041	1.03	U76558	0.265

TC234060	1.06	TC234060	0.278

TC247734	1.08	TC248038	0.298

AF251264	1.11	AF251264	0.325

AF475127	1.14	AF475127	0.334

TC248640	1.17	AF021243	0.337

D13147	1.20	TC247734	0.360

NP114267	1.23	AY587265	0.382

U76558	1.26	TC51135	0.396

AF244997	1.30	U76895	0.419

NP114282	1.33	X14348	0.421

AY587265	1.36	AJ635206	0.512

UF76895	1.42	AY727927	0.529

UF76745	1.47	U76475	0.532

AY727927	1.52	NP114267	0.569

AF021243	1.59	NP114282	0.678

NormFinder algorithm, which is based on a variance estimation approach [25] and ranks the genes according to their stability under a given experimental conditions, was used to asses the gene stability. Table 3 presents the ranking of the 24 genes based on their expression stability. The most stable gene *TaWIN1 *(AB042193) is encoding for a 14-3-3 like protein which had also a stability value (M) estimated by geNorm lower than 1.

Although the order of the top-ranked candidate genes estimated by NormFinder was not the same as the one calculated by geNorm, the genes with accession numbers AJ457980.1, AB042193, TC234027, AY456122.1, and AL827977.1 were found as the best consensus considering their gene rankings in both methods.

### Evaluation of stability of selected reference genes in different winter wheat varieties

**Figure 3:**
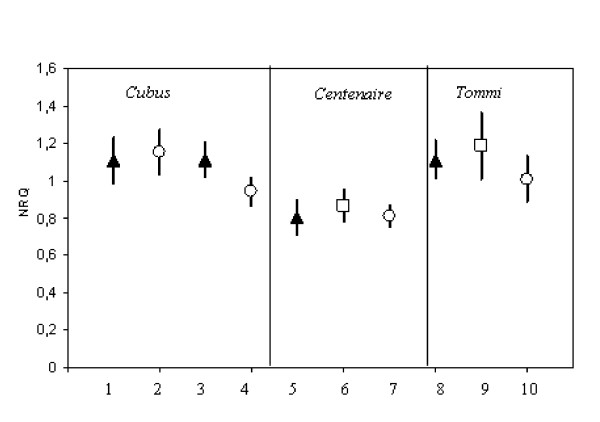
**Normalized relative quantities (NRQ) of the *TaFNRII *gene estimated in winter wheat flag leaves of three varieties grown under different agricultural production systems**. Symbols and bars represent the mean and confidence limits at 95% level, respectively. X-axis: Lanes 1-4: cv. Cubus collected in 2006; lanes 5-7: cv. Centenaire; collected in 2008; lanes 8-10: cv. Tommi; collected in 2008; triangle: conventional agriculture; circle: organic agriculture; square: no nitrogen applied.

**Figure 4:**
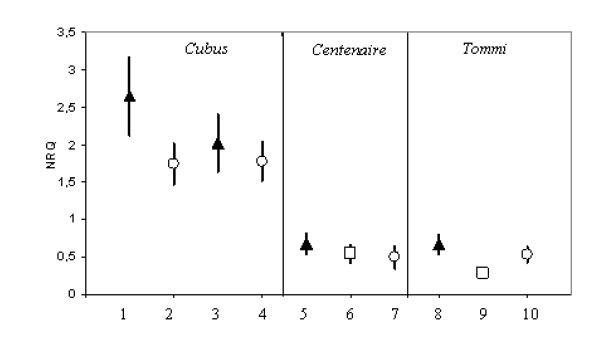
**Normalized relative quantities (NRQ) of the *rrn26 *gene estimated in winter wheat flag leaves of three varieties grown under different agricultural production systems**. Symbols and bars represent the mean and confidence limit at 95% level, respectively. X-axis: Lanes 1-4: cv. Cubus collected in 2006; lanes 5-7: cv. Centenaire collected in 2008; lanes 8-10: cv. Tommi collected in 2008; triangle: conventional agriculture; circle: organic agriculture; square: no nitrogen applied.

To evaluate the robustness of the selected reference genes tested in cv. Cubus two other wheat varieties were investigated. In addition to the samples of winter wheat cv. Cubus grown in 2006 under two different environments, RT-qPCR analysis was also conducted in 60 samples of cv. Tommi and Centenaire, collected in 2008 from organic and conventional farming systems and no nitrogen fertilization treatment located in a third environment. The quality of the reference genes tested was automatically determined by the calculation of M values. The stability of the genes *ACT2 *(actin 2; TC234027), *TaFNRII *(ferredoxin-NADP(H) oxidoreductase; AJ457980.1), *rrn26 *(AL827977.1), and *CYP18-2 *(Cyclophilin A; AY456122.1), have been confirmed on cv. Centenaire and cv. Tommi. In this case, in all varieties tested, M was 0.6 for both TC234027 and AJ457980.1 with a CV of 21% and 20%, respectively and 0.95 with a CV 40% for AL827977.1, and 1.15 for AY456122.1 with a CV of 55%. As an example, Figure 3 shows the stability of the reference gene *TaFNRII *in all winter wheat varieties tested grown under different agricultural production systems and environments. However, the level of gene expression of gene *rrn26 *differs among cv. Cubus and cv. Tommi or cv. Centenaire, which makes it unsuitable for normalization in case different varieties are analysed together (Figure 4). Nonetheless it is not fully certain that this variation is linked to the genetic background of wheat as the cv. Cubus was sampled in 2006 while the other two varieties were collected in 2008. Since the purpose was to select stable reference genes in wheat leaves, which should be consistently independent of the variety or the treatment, this gene was considered as being not suitable for gene expression in future gene expression analysis.

## Discussion

The use of gene expression profile in organic farming comes mainly from the large interest of researchers to identify potential diagnostic genes that can improve the nitrogen use efficiency of wheat [17,31,32]. Moreover if specific gene expression is diagnostic for use of organic sources of nitrogen fertiliser they may therefore have useful applications in defining the differences between organically and conventionally grown wheat. Indeed, protection of the rights of consumers, genuine food processors, and prevention of fraudulent or deceptive practices and the adulteration of food is an important and challenge facing the food industry [33]. Recently attempts were made to apply RT-qPCR aiming to solve food authentication problems [23,34]. This approach is however emerging and requires further experimental evaluation [35]. Our study contributes to these challenges by identifying reliable reference genes for normalising gene expression in winter wheat flag leaves.

In wheat flag leaves, previously transcriptome analysis has shown that several genes are up-regulated such as transcription factors (WRKY) or down-regulated such as genes from hormone pathways or carbohydrate metabolism [36]. In general the gene expression profile is heterogeneous and complex and depends on the tissues analysed, developmental stages of the plant and environmental conditions. In this study, the expression of several traditional used reference genes and new set of genes was measured in wheat flag leaves grown under organic and conventional agricultural production systems. The reference genes will be further use in a large scale gene expression analysis for identification of changes in transcripts accumulation associated with a certain agricultural production system. Transcription is a very complex and versatile process that responds to biotic and abiotic factors, with some genes being highly inducible while others are constitutively expressed. The environmental conditions might influence the gene expression, but in this experiment we were interested to search and select such genes, which were stable expressed without monitoring these factors as usual performed in controlled environmental condition. For that reason, a first set of winter wheat flag leaves were collected in 2006 from two Belgian locations and in each of them two paired open fields (certified organic *vs*. conventional) were sampled while a second set of winter wheat flag leaves were collected in 2008 from a third Belgian location.

Because a universal reference gene does not exist and the genes are regulated differently depending on specific conditions it is necessary to search for suitable genes in every experimental design situation [10]. However, to achieve reliable results, it is essential to determine the expression stability of several candidate reference genes to normalize the variation given by RNA sample quality, reverse-transcription efficiency, etc. In previous gene expression studies several genes involved in cellular processes such as ubiquitin and cyclophilin or in cell structures such as actins and tubulins were the most used genes for normalization of PCR reaction, but several studies have shown that the expression of such genes is not as independent of experimental condition as expected [37-40]. Utilization of a single reference gene, reported before in many gene expression studies [41,42], may lead to an inaccurate biological conclusion [43]. Therefore, utilization of additional reference genes can give more accurate and reliable results [25,44-46].

Several approaches have been proposed to identify reference genes in wheat such as all genome-wide transcriptome or microarray analysis [7,47], but generally the experimental design differs starting with the type of biological material used, experimental conditions, developmental stages of plants and finally with the selection of software tools (e.g. geNorm, NormFinder or BestKeeper) used to examine the stability of gene expression [4,25,48]. All methods aim at defining the "most stable" gene from a set of genes, wherein "expression stability" is referred to as the least variation of constitutive expression levels in the group of samples analysed [49]. There is no clear answer which strategy should be used to address the issue of reference gene expression stability for a specific experimental setup. Nonetheless, studies comparing several algorithms obtained similar rankings of the reference genes [4,50]. Recently, using microarray approach on Triticeae species, Gimenez et al. identified six candidate genes which showed stable expression in their experimental design. Based on their analysis, the ranking of the reference genes in terms of stability is not identical in all the cases assays and the best choice of the reference genes would differ upon the species being compared [51].

In the present study, the selection of genes was based on the hypothesis that differential level of gene expression could be expected between organically and conventionally grown wheat due to different crop husbandries (e.g. phytosanitary or fertiliser treatments; data available on request) and a valid internal control to normalise the gene expression is needed. The gene expression stability was evaluated on several commonly used reference genes encoding for actins, tubulins, elongation factor 1 beta or cyclophilin, and other genes such as 18S ribosomal protein or genes chosen from different metabolic functions such as, the defence/resistance genes, RuBisCO small and large subunits, low affinity nitrate transporter or ADP-glucose pyrophosphorylase.

Tubulins (*Tubb2 *and alpha-tubulin), and elongation factor 1 beta were not stably expressed in wheat flag leaves under our experimental conditions. Several studies conducted in grapes, sugar cane and potato showed that the *TUB *gene performed poorly as a reference gene [11]. On the other hand, ubiquitin UBQ5 and elongation factor 1 alpha were the most stable genes expressed in rice [50]. However, several potential reference genes seem to be regulated differentially in different plant species or under different environmental conditions.

In previous gene expression analyses, *18S *was the most often used gene for normalization of PCR reactions [52], but some other studies showed that the expression of this gene was regulated [26,53] and transcript abundance varied from sample to sample. In this study, we have included a *18S *subunit ribosomal protein (AF475127.1) which displayed a median C_q _of 18.7 with CV of 11.1% across all samples assayed (Figure 1) and had low rank stability values estimated by both geNorm and NormFinder algorithms (Table 3).

Beta-actin was also used as a reference gene in previous studies, but increasingly it has become apparent that its expression level is not as stable as had been expected [39]. For example, the poor performance of *ACT2 *as reference gene has been already showed in potato, rice and soybean [54-56], while the *ACT2/7 *was seen to be rather variable in *Arabidopsis *[57]. However other studies reported that actins are stably expressed such as *ACT1 *in rice seeds or *ACT11 *in soybean tissues at various developmental stages, varied photoperiodic treatments, and a range of soybean cultivars, which makes them suitable for PCR normalization [11,58]. Among the five actin-like genes tested in our study for their stability, the *ACT2 *gene (TC234027) was expressed most stably in all wheat flag leaves samples assayed. The other actins were variably expressed in wheat flag leaves and this variation could be related to the developmental stage of the wheat leaf. This is in concordance with several studies in *Arabidopsis *showing that several actins genes are divergent and differentially expressed in different tissues [57].

Using the geNorm approach the optimal number of reference genes was determined by pair-wise variation analysis of normalization factors with a cut-off of 0.15 [26]. This value is not absolute, and, in some cases, may not be achieved [59]. In the current study, V_3/4 _= 0.149 suggesting that three reference genes should be sufficient for normalization of RT-qPCR data from winter wheat cv. Cubus flag leaves: *TaFNRII *encoding for the ferredoxin-NADP(H) oxidoreductase (AJ457980.1), an important enzyme which belongs to a large family of flavoproteins founded in higher plants [60] and *ACT2 *were the most stable reference genes in both organic and conventional wheat flag leaves, followed by *rrn26*, a wheat mitochondrial gene that encodes the mitochondrial large-subunit rRNA 26S (AL827977.1) but its function in wheat is still unknown. In addition of these three genes that were also top-ranked by NormFinder, two extra genes with accession numbers AY456122.1 and AB042193 were most consistently stably expressed (Table 3).

Cyclophilins, as the cyclophilin A gene *CYP18-2 *(AY456122.1), are enzymes that catalyse the rate-limiting step of peptidyl-prolyl *cis-trans *isomerisation. They are known to be up-regulated during wheat endosperm development and suggested to be important for folding of the storage proteins, thus influencing wheat quality [61]. However this gene was stably expressed in wheat flag leaves sampled in organic and conventional fields considering that this organ allows for an efficient translocation of assimilates and contribute to the final grain nitrogen level.

*TaWIN1 *(AB042193) encodes a 14-3-3 protein that binds to a wheat protein kinase (WPK4) which most likely is responsible for controlling the nitrogen metabolic pathway; nitrate reductase is inactivated by phosphorylation followed by 14-3-3 protein binding [62].

Two additional winter wheat varieties (i.e. cv Tommi and Centenaire), included here as a validation group, were grown in 2008 under organic, conventional and without nitrogen fertilisation conditions in order to test the stability of the selected three reference genes. Results showed that three genes, *ACT2, TaFNRII*, and *CYP18-2 *but not *rrn26*, remained the most stable genes in flag leaves in all varieties tested. In a microarray study performed in wheat grains grown under organic and inorganic treatments, it has been suggested that the gene *rrn26 *(AL827977.1) can be a diagnostic gene for organically grown grains [23]. The gene displayed a higher gene expression when grains were grown on farmyard cattle manure. In our study, we observed that the expression of this gene in flag leaves could differ from one cultivar to another while the level of expression seemed to be not influenced by the environments. This indicates the complexity of the transcriptional event in plant developmental stages and that some genes which can be used as a reference gene in certain organ or experimental conditions may not be suitable to normalize gene expression under other condition [43]. Therefore, it is necessary to determine the most stable genes under different experimental conditions [63], which in our case means various wheat varieties and environmental conditions including existing variability in the agricultural production systems.

Moreover these results confirm the recent recommendations to select not only stably expressed genes in a pilot experiment with representative samples but also to assess their expression stability in the final experiment [46].

## Conclusion

In conclusion, we have identify the most three stable and uniformly expressed genes, suitable for normalization of RT-qPCR results in three winter wheat cultivars grown in open fields under different well recorded crop husbandries at three locations and collected in different years. These data provide a new set of reference genes that can be useful for further gene expression studies in winter wheat flag leaves as for instance those related to the improvement of nitrogen use efficiency for cereal production.

## Competing interests

The authors declare that they have no competing interests.

## Authors' contributions

GNT carried out the molecular genetic study on cv Tommi and Centenaire and drafted the manuscript. APB developed the method and carried out the molecular study on cv Cubus. FCR contributed to the statistical analysis. AM conceived the design of the study, performed the statistical analysis and helped to draft the manuscript. All authors read and approved the final manuscript.

## Supplementary Material

Additional file 1 **Primer and probe sequences used to quantify the expression of the selected traditional reference candidate genes by real-time PCR**. Click here for file

Additional file 2 **Primer and probe sequences used to quantify the expression of the selected new set of candidate genes by real-time PCR**. Click here for file
