# Supplementary material for: Reference genes for gene expression studies in wheat flag leaves grown under different farming conditions
Source: BMC Res Notes. 2011 Sep 27;4:373. doi: 10.1186/1756-0500-4-373 (PMC3193821; doi:10.1186/1756-0500-4-373)
Supplement: Additional file 1 — Primer and probe sequences used to quantify the expression of the selected traditional reference candidate genes by real-time PCR. [file 1756-0500-4-373-S1.DOC]

**Additional file 1.**

File format: doc

**Title: Primer and probe sequences used to quantify the expression of the selected traditional reference candidate genes by real-time PCR.**

| **Gene Identification Nb^1^**  **(mRNA)** | **Forward primer Seq** | **Probe Seq**  **(MGB/TaqMan)** | **Reverse primer Seq** | **PCR efficiency** | **R2 values** |
| --- | --- | --- | --- | --- | --- |
| AF021243 | AATCAACCTTCTCAAGGTGGACTT | AAGGAAATTGAGGCCATTGCAGGTGA | TCTCAGACACTTCTTTTGCTTGCT | 1.97 | 0.9875 |
| AF475127.1 | CCGCTCAATCGCAGAATGT | ACGGTGGTCGTCGAAAACCCA | AATTTCCCCTTGTCATCTACTGTCA | 2.00 | 0,9929 |
| AY049041 | GTCGGCCGCCTCGAA | CCTTCCCAACGGGCGGTGG | TTAAGTCGTCTGCAAAGGATTCAG | 2.00 | 0,9860 |
| AY456122 | GATCTCCGTGGTTGGTTTAGGA | TTTTAGGTGGACTCTTTTGGCC | CGCCGGACACAGATCCA | 1.97 | 0,9995 |
| D13147^2^ | TGCCAAGCCTGCAAAGAAG | AAGAAAGTGGCAAATCCTCCGTCCTCA | CGTCCCATGGCTTGATGTC | 1.97 | 0,9964 |
| TC234027 | CAAATCATGTTTGAGACCTTCAATG | TGCTAGTGGACGCACAA | ACCAGAATCCAACACGATACCTG | 1.88 | 0,9999 |
| TC234060 | GATCATGTTCGAGACCTTCAACTG | TCTACGCCAGCGGC | GACGATGCCGGTGGTCC | 1.83 | 0,9996 |
| TC247734 | CAGATTATGTTTGAGACTTTCAATGTTC | ATGCAAGTGGTCGTACTA | CCAGAGTCGAGAACAATACCGG | 2.00 | 0,9920 |
| TC248038 | TCATGTTTGAGACATTCAGTGTTCC | TGCTAGTGGGCGTACCA | CCGGAATCCAAGACAATACCAG | 1.90 | 0,9990 |
| TC248640 | CATGTTCGAGACATTCAATGCC | TCTATATGCCAGTGGTCGTA | CCAGAATCAAGCACAATACCTGTAG | 1.97 | 0,9966 |
| U76558 | CCAGTGTCGTCGAGGTCTTCTC | CGCATCGACCACAAGTTTGACCT | GGCACGCTTGGCGTACAT | 2.00 | 0,9950 |
| U76745 | CGAGGAGGGCGAGTACGA | AGGAGCCTGAGGAGGACATGTAAGGT | AGCAAAGCACGACATGGACAT | 2.00 | 0,9966 |
| U76895 | CCTCCTGTGGCTTGGATGA | TCGAGGTTGCGTCTTGTGCT | CTAGCCGTAGCAAATTACAAAACG | 2.00 | 0,9978 |

^1^ http://www.ncbi.nlm.nih.gov/UniGene or TIGR Gene Indices (http://compbio.dfci.harvard.edu/tgi/plant.html)"

^2^ TaqMan probe.

R^2^, correlation coefficient of the slope of the standard curve
