# Supplementary material for: Reference genes for gene expression studies in wheat flag leaves grown under different farming conditions
Source: BMC Res Notes. 2011 Sep 27;4:373. doi: 10.1186/1756-0500-4-373 (PMC3193821; doi:10.1186/1756-0500-4-373)
Supplement: Additional file 2 — Primer and probe sequences used to quantify the expression of the selected new set of candidate genes by real-time PCR. [file 1756-0500-4-373-S2.DOC]

**Additional file 2.**

File format: doc

**Title: Primer and probe sequences used to quantify the expression of the selected new set of candidate genes by real-time PCR.**

| **Gene Identification Nb^1^**  **(mRNA)** | **Forward primer Seq.** | **Probe Seq**  **(MGB)** | **Reverse primer Seq** | **PCR efficiency** | **R2 values** |
| --- | --- | --- | --- | --- | --- |
| AB042193 | CATCACTACCTCTGTTTAATTATGCACTT | AAGTTTCTGGGCTATGTTTTTCTGGTGCTGAT | CACGAATCCAGCCAGCATT | 1,95 | 0,9981 |
| AF244997 | CGAGGCCGCGAAGAGAT | AGCAATAAAAAAGGAGTGCCATGCAAGGC | AGGGAGAAAAAGGGTGGAGAAG | 2,00 | 0,9884 |
| AF251264 | TGAGAAGGAAGGACGCTTTCATG | CTGCAGCGTGTTCTAC | TCTGCTTTATCGAGCGTTGCA | 1,90 | 0,9979 |
| AJ457980 | CACCGGCCCAGTGATCTT | AACCCGGCTTCAGGTCACAGAGG | AAGGGCGTCTGCTCCAACT | 1,89 | 0,9955 |
| AJ635206 | CGACTACTTGATCCGCTCCAA | CCCTGCCTCGAGTTCA | ACGGAAGACGAAACCAACCTT | 1,96 | 0,9995 |
| AL827977 | TGAGAAGGAAGGACGCTTTCATG | CGGAGATCCATGGTATCACAGAAAA | ACCATGGCCTTTCGCC | 1,97 | 0,9993 |
| AY587265 | TGGCTGTTCCGATGAGCAT | ATGTGGCAAGGACCCCAGTACTTC | TCGGCGACGCCTAGCA | 1,85 | 0,9978 |
| AY727927 | TTTCCCATGCAGCAAGTTCA | CAAGCTCTGCATCGACTCGGG | GTTTTATTATTTCCGCCGATGATAC | 1,97 | 0,9993 |
| NP_114282.1 | GTGGGTTCTCCTCCAAGCA | CTCCATGGCATCCTTC | GCAAACGGTGACACCAGATATATATACAA | 1,95 | 0,9999 |
| NP_114267.1 | CAGCAGCTAATCGAGTGGCTTTA | CCCTTCGTTACGAGCTTG | TCATTACCTTCGCGAGCAAGATC | 1,95 | 0,9999 |
| X14348 | GGCGTCAGAAGGCTTCCA | ATCCGGTCCGGTATCACGGTT | CGCAATCACCGAGTTCTTCA | 2,00 | 0,9997 |

^1^ http://www.ncbi.nlm.nih.gov/UniGene;

R^2^, correlation coefficient of the slope of the standard curve
